# The Contrasting Delayed Effects of Transient Exposure of Colorectal Cancer Cells to Decitabine or Azacitidine

**DOI:** 10.3390/cancers14061530

**Published:** 2022-03-16

**Authors:** Alicja Pawlak, Kinga Chybicka, Ewa Zioło, Leon Strządała, Wojciech Kałas

**Affiliations:** Ludwik Hirszfeld Institute of Immunology and Experimental Therapy, Polish Academy of Sciences, R. Weigla 12, 53-114 Wroclaw, Poland; al.paw.pub@gmail.com (A.P.); kinga.chybicka@hirszfeld.pl (K.C.); ewa.ziolo@gmail.com (E.Z.); leon.strzadala@hirszfeld.pl (L.S.)

**Keywords:** azacitidine, decitabine, colon cancer, epigenetic drug, cellular senescence, chemotherapy, vidaza

## Abstract

**Simple Summary:**

Decitabine and azacitidine are cytosine analogs representing the class of drugs interfering with DNA methylation. Due to their molecular homology and similar clinical application these drugs are viewed as interchangeable. Despite their unique epigenetic mechanism of action, the studies of the prolonged activity of decitabine and azacitidine are rare. Our head-to-head comparison revealed profound differences in the activities of decitabine and azacitidine important in their anti-cancer potential and clinical application. We show that azacitidine, despite significant immediate toxicity, has negligible long-term effects. Contrary, decitabine, which does not exert initial toxicity, profoundly worsened the condition of the cancer cells over time. The effects of decitabine need a relatively long time to develop. This property is crucial for the proper design of studies or therapy involving decitabine. It undermines opinion about the similar therapeutic mechanism and interchangeability of decitabine and azacitidine.

**Abstract:**

(1) Background: Decitabine and azacitidine are cytosine analogues representing the class of drugs interfering with DNA methylation. Due to their molecular homology and similar clinical application, both drugs are often regarded as interchangeable. Despite their unique mechanism of action the studies designed for observation and comparison of the prolonged activity of these drugs are rare. (2) Methods: The short-time (20–72 h) and long-term (up to 20 days) anti-cancer activity of decitabine and azacitidine has been studied in colorectal cancer cells. We observe the impact on cell culture’s viability, clonogenicity, proliferation, and expression of *CDKN1A, CCND1, MDM2, MYC, CDKN2A, GLB1* genes, and activity of SA-β-galactosidase. (3) Results: Decitabine has much stronger anti-clonogenic activity than azacitidine. We show that azacitidine, despite significant immediate toxicity, has negligible long-term effects. Contrary, decitabine, which does not exert initial toxicity, profoundly worsened the condition of the cells over time. On the 13th day after treatment, the viability of cells was decreased and proliferation inhibited. These functional changes were accompanied by up-regulation of expression *CDKN1A, CCND1, and CDKN2A* genes and increased activation of SA-β-galactosidase, indicating cellular senescence. (4) Conclusions: Our head-to-head comparison revealed profound differences in the activities of decitabine and azacitidine important in their anti-cancer potential and clinical application. The effects of decitabine need relatively long time to develop. This property is crucial for proper design of studies and therapy concerning decitabine and undermines opinion about the similar therapeutic mechanism and interchangeability of these drugs.

## 1. Introduction

It is generally accepted that, apart from genetic mutations in pro-oncogenes or tumor suppressor genes, epigenetic modifications may drive the oncogenic transformation [1,2]. The aberrant pattern of DNA methylation in neoplastic cells is often manifested as global hypomethylation accompanied by local hypermethylation of CpG islands. The latter event leads to the repression of expression of many genes, including cancer suppressor genes [3,4].

Azacitidine, along with its deoxyribose counterpart decitabine, were initially discovered as “classic” anti-metabolite drugs and were extensively tested for anti-cancer activity [5,6,7]. Twenty years later, the discovery of their potential to inhibit DNA methylation drew broader attention to azacitidine and decitabine, again. Substitution of the 5th carbon of cytosine with nitrogen prevents attachment of a methyl group to cytosine by methyltransferases during the DNA methylation reaction. It results in permanent locking of enzyme-DNA complexes. Finally, due to the sequestration of methyltransferases, global inhibition of DNA methylation occurs during subsequent rounds of DNA replication [8,9,10,11]. According to their potent myelosuppressive activity observed in early clinical trials, both drugs have been tested and registered for the treatment of myelodysplastic syndromes (MDS). The 75 mg/m${}^{2}$/day for 3–7 days of azacitidine or 20 mg/m${}^{2}$/day for 3–5 days of decitabine are the recommended regiments [12]. Thus, decitabine and azacitidine became founding members of a novel class of drugs interfering with DNA methylation and with the ability to modify epigenetic information. Both drugs are frequently regarded as interchangeable, mainly due to their molecular homology and analogical clinical recommendations [13,14,15,16,17,18].

Recently, a growing number of studies on decitabine and azacitidine suggest separate mechanisms of action of these drugs [19]. Additionally, studies of global gene expression in the context of inhibition of DNA methylation provide indirect genetic evidence of the disparate mechanisms of action of decitabine or azacitidine [20,21,22]. For example, DNA sequencing analysis of acute myeloblastic leukemia revealed that only 6–12% of genes induced by decitabine or azacitidine overlap. Similarly, in non-small cell lung cancer cell line A549, distinct groups of genes were up-regulated upon 48h treatment with decitabine or azacitidine [22]. The further analysis revealed that azacitidine down-regulated genes are responsible for cell cycle progression and proliferation, while decitabine up-regulated genes that take a part in cell differentiation. Both drugs enter the cell by nucleoside transporters and are transformed into their three-phosphorylated derivates [23]. All of decitabine, the deoxyribose derivate of 5-azacytosine, can be incorporated into DNA during DNA replication. There is no evidence for decitabine incorporation into RNA. Azacitidine, the ribose derivate of 5-azacytosine, is accordingly to bonded sugar, primarily incorporated into RNA. Only 10–35% of azacitidine dose is transformed into deoxy- derivate, which could be incorporated into DNA [12,24]. Regardless that most of the azacitidine molecules are built-in into RNA, its RNA-related effects were rarely examined. Recently, it has been suggested that RNA-mediated chromatin disorganization could be the primary for anti-leukemic activity of azacitidine [25]. The another report identified inhibition of nonsense-mediated RNA decay by azacitidine as a reason for its cytotoxicity. Significantly, wide range of other cytidine analogs, including decitabine, have no such activity [26]. Emerging differences resulted in direct and retrospective studies comparing the clinical efficacy of decitabine and azacitidine. A recent retrospective analysis by Ma and Ge indicates a higher overall response in AZA than DAC-treated, with a lower frequency of grade 3/4 cytopenia in AML and HR-MDS patients [27]. On the other hand, the Jabbour et al. and Hu et al. studies of MDS indicated better overall response for patients treated with DEC than AZA, but on low-dose regiments [14,28]. Differences in clinical efficacy alongside some of its molecular aspects were comprehensibly reviewed [12,19,27,29,30,31]. There are still attempts to extend the application of decitabine and azacitidine to solid tumors, but their efficiency in mono-therapy is unsatisfactory [15,32]. For that reason, 5-aza-modified cytosine derivatives were tested in combination therapy [33,34]. There are in vitro studies demonstrating that epigenetic drugs could successfully serve as chemo-sensitizers [34,35]. We also have shown that both decitabine and azacitidine can sensitize the wide panel of colorectal cancer cells to sequential treatment with various inhibitors of topoisomerases [36]. The nature of such sensitization is very diverse. It can vary from restoring sensitivity to intrinsic- (HA1004) or extrinsic- (FK506) apoptosis pathways, reprogramming of malignant cells [37,38], to rise of expression of tumor-specific antigens facilitating the anti-cancer immune response [32].

We want to underline that most of the studies of decitabine or azacitidine activities are up to 3 days long. In such a short time, the immediate stress response to the drug may conceal the epigenetic interference with the cells, which, in theory, should be prolonged or even permanent [8,9,39,40]. Thus, in our opinion, studies of the activities of epigenetic drugs should be extended in time. Regarding growing evidence of the disparate mechanism of decitabine and azacitidine, we have decided to perform a comprehensive head-to-head comparison of the long-term effects of these two drugs. Such knowledge is crucial for the reasonable use of these epigenetic drugs in science studies and cancer therapy.

## 2. Materials and Methods

### 2.1. Cell Culture

The human colorectal cancer cell lines DLD-1, HCT116, HT-29, SW948, and LoVo (Institute of Immunology and Experimental Therapy, IIET, Wrocław, Poland) were cultured in DMEM (IIET) supplemented with 4.5 g/L D-glucose (POCH), 2 mM L-glutamine (Sigma-Aldrich, Merck KGaA, Darmstadt, Germany) 10 mM HEPES (Sigma-Aldrich), 40 µM β-mercaptoethanol (Sigma-Aldrich), 10% fetal bovine serum (FBS; Gibco, Thermo Fisher Scientific, Waltham, MA, USA). The human colorectal cancer cell line RKO (#CRL-2577, ATCC) was cultured in EMEM (IIET) supplemented with essential and nonessential amino acids, 1 mM sodium pyruvate (Serva), 10 mM HEPES, 40 µM 2β-mercaptoethanol, 10% FBS. The human normal small intestine epithelial cell line FHs 74 Int (#CCL-241, ATCC) was cultured in Hybri-Care medium (ATCC) supplemented with 1.5 g/L sodium bicarbonate (POCH), 35 ng/mL epidermal growth factor (Sigma-Aldrich), 10% FBS. All media were supplemented with Antibiotic Antimycotic Solution (Sigma-Aldrich). The cells were cultured under standard conditions (37 °C, 95% humidity, 5% CO_2_) and detached from culture vessels by treatment with trypsin-EDTA solution (0.125%; IIET). The following culture vessels were used in this study: 96-well plates (0.32 cm${}^{2}$; Corning, New York, NY, USA), 24-well plates (1.9 cm${}^{2}$; Corning), 6-well plates (9.5 cm${}^{2}$; Corning), dishes (21 cm${}^{2}$; Sarstedt, Nümbrecht, Germany), microscope chamber slides (0.7 cm${}^{2}$; Thermo Scientific, Waltham, MA, USA).

### 2.2. Decitabine and Azacitidine

Decitabine (5-aza-2′-deoxycytidine; #11166, Cayman Chemical, Ann Arbor, MI, USA) and azacitidine (5-azacytidine; #11164, Cayman Chemical) were dissolved in dimethyl sulfoxide (DMSO; Sigma-Aldrich) and further diluted in culture medium immediately before use.

### 2.3. Clonogenic Assay

**Figure A1:**
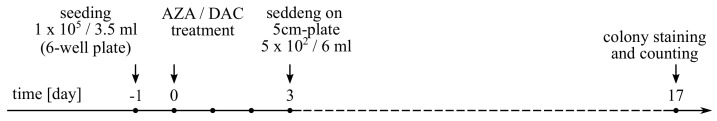
Detailed scheme of treatments for colony formation assay.

The DLD-1 cells were seeded at a density of 1 × 10${}^{5}$ cells/3.5 mL (9.5 cm${}^{2}$), the next day (day 0) decitabine (0.1–1 µM) or azacitidine (0.4–4 µM) was added, 3 days later the cells were seeded at a density of 5 × 10${}^{2}$ cells/6 mL (21 cm${}^{2}$) on dishes pre-coated with poly-L-lysine (150–300 kDa; Sigma-Aldrich) and incubated for 14 days under standard cell culture conditions to count the colonies on the last day (Figure A1). The colonies were fixed, permeabilized, and stained with 1% crystal violet (Sigma-Aldrich) in ethanol (POCH, Avantor Performance Materials Poland S.A., Gliwice, Poland) for 30 min at 4 °C. The results are shown as a percentage of control (untreated cells) and as a number of colonies.

### 2.4. Cell Viability Assay (MTS Assay)

**Figure A2:**
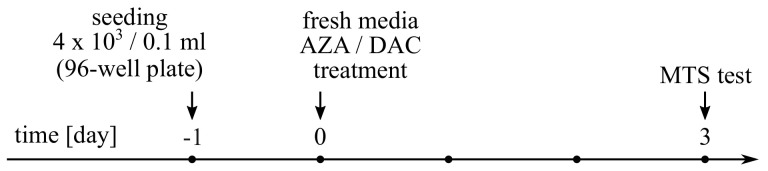
Detailed scheme of treatments for 3 day long MTS assay.

The cell treatments in 3-day experiments were as follows: The DLD-1, HCT116, HT-29, SW948, LoVo, and RKO cells were seeded at a density of 4 × 10${}^{3}$ cells/0.1 mL (0.32 cm${}^{2}$), the next day (day 0) decitabine (1–50 µM) or azacitidine (1–50 µM) was added, and cell viability was measured after 3 days. The FHs 74 Int cells were seeded at a density of 8 × 10${}^{3}$ cells/0.1 mL (0.32 cm${}^{2}$), the next day (day 0) decitabine (1 µM) or azacitidine (4 µM) was added, and cell viability was measured after 3 days (Figure A2). The cell treatments in 13-day experiments were as follows: The DLD-1, HT-29, and RKO cells were seeded at a density of 1 × 10${}^{5}$ cells/3.5 mL (9.5 cm${}^{2}$), the next day (day 0) decitabine (1 µM) or azacitidine (4 µM) was added, then the cells were cultured for 10 days (first passage after 3 days) and seeded at a density of 8 × 10${}^{3}$ cells/0.1 mL (0.32 cm${}^{2}$) to measure cell viability on day 13. The FHs 74 Int cells were seeded at a density of 3 × 10${}^{4}$ cells/1 mL (1.9 cm${}^{2}$), the next day (day 0) decitabine (1 µM) or azacitidine (4 µM) was added, then the cells were cultured for 7 days (first passage after 3 days) and seeded at a density of 8 × 10${}^{3}$ cells/0.3 mL (0.32 cm${}^{2}$) to measure cell viability on day 13 (Figure A4). Cell viability was measured using CellTiter 96 AQueous One Solution Cell Proliferation Assay (Promega, Madison, WI, USA) according to the manufacturer’s protocol. The results are shown as a percentage of control (DMSO-treated cells). The IC${}_{50}$ values were calculated in GraphPad Prism 7.03 (GraphPad Software).

### 2.5. Western Blotting

**Figure A3:**
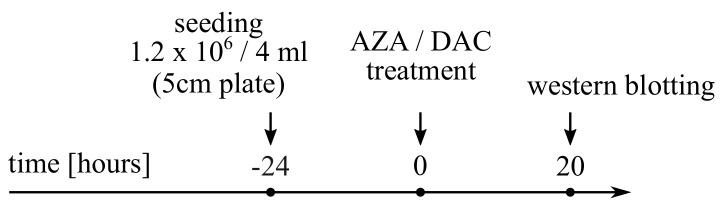
Detailed scheme of treatments for western blotting.

**Figure A4:**
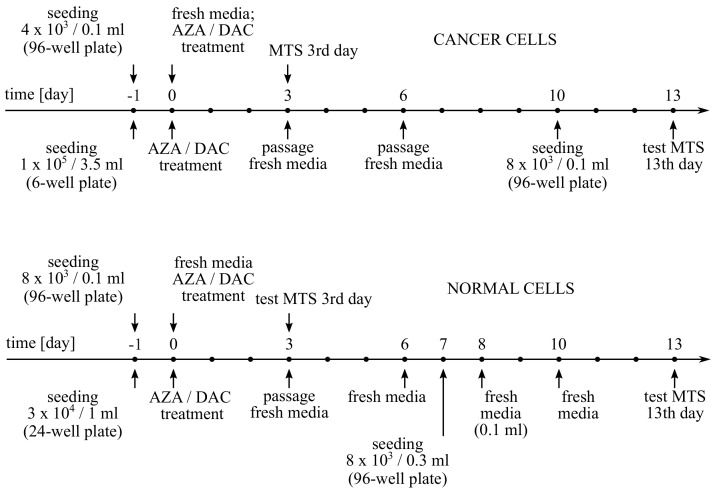
Detailed scheme of treatments for 13 day long MTS assay.

The cell treatments in CHOP expression experiments were as follows: The DLD-1 and HT-29 cells were seeded at a density of 1.2 × 10${}^{6}$ cells/4 mL (21 cm${}^{2}$), the next day (day 0) decitabine (1–10 µM) or azacitidine (1–10 µM) was added, and CHOP expression was measured after 20 h. The cell treatments in p21 expression experiments were as follows: The DLD-1 cells were seeded at a density of 3.5 × 10${}^{5}$ cells/4 mL (21 cm${}^{2}$), the next day (day 0) decitabine (1 µM) or azacitidine (4 µM) was added, then the cells were cultured for 20 days (first passage after 3 days) to measure p21 expression on the last day (Figure A3 and Figure A7B). The cells were lysed with RIPA buffer (IIET) supplemented with SigmaFAST Protease Inhibitor Cocktail (Sigma-Aldrich) and sonicated for 10 s by Sonopuls HD 2070 ultrasonic homogenizer (Bandelin, Berlin, Germany). Total protein concentration was measured using Pierce BCA Protein Assay Kit (Thermo Scientific, Waltham, MA, USA) according to the manufacturer’s protocol. Proteins were resolved by 10–12% SDS-PAGE and transferred to PVDF membrane (0.45 µm pore size; Merck Millipore, Darmstadt, Germany). The membrane was blocked with 1% casein (Sigma-Aldrich) overnight at 4 °C and incubated with horseradish peroxidase (HRP)-conjugated anti-actin antibody (1:3000; #sc-1615, Santa Cruz Biotechnology) or HRP-conjugated anti-p21 antibody (1:2000; #sc-6246, Santa Cruz Biotechnology, Dallas, TX, USA) for 1 h at room temperature (RT), or with unconjugated anti-CHOP antibody (1:1500; #2895, Cell Signaling Technology, Danvers, MA, USA) overnight at 4 °C. In the latter case, the membrane was subsequently incubated with HRP-conjugated anti-mouse antibody (1:2500; #P0447, Dako, Santa Clara, CA, USA) for 1 h at RT. The protein of interest was detected using SuperSignal West Dura Extended Duration Substrate (Thermo Scientific, Waltham, MA, USA) according to the manufacturer’s protocol. Actin was used as a loading control.

### 2.6. Microscopic Images

**Figure A5:**
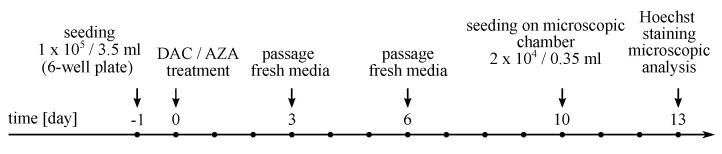
Detailed scheme of treatments for microscopic staining.

The DLD-1 cells were seeded at a density of 1 × 10${}^{5}$ cells/3.5 mL (9.5 cm${}^{2}$), the next day (day 0) decitabine (1 µM) or azacitidine (4 µM) was added, then the cells were cultured for 10 days (first passage after 3 days) and seeded at a density of 2 × 10${}^{4}$ cells/0.35 mL (0.7 cm${}^{2}$) to take microscopic images on day 13 (Figure A5). The cells were fixed with 4% paraformaldehyde (Sigma-Aldrich) for 30 min under standard cell culture conditions and stained with 2 µM PureBlu Hoechst 33342 (Bio-Rad) for 15 min at RT. Microscopic images were taken by Olympus IX81 inverted fluorescence microscope (Olympus, Tokyo, Japan).

### 2.7. Flow Cytometry—Cell Proliferation

**Figure A7:**
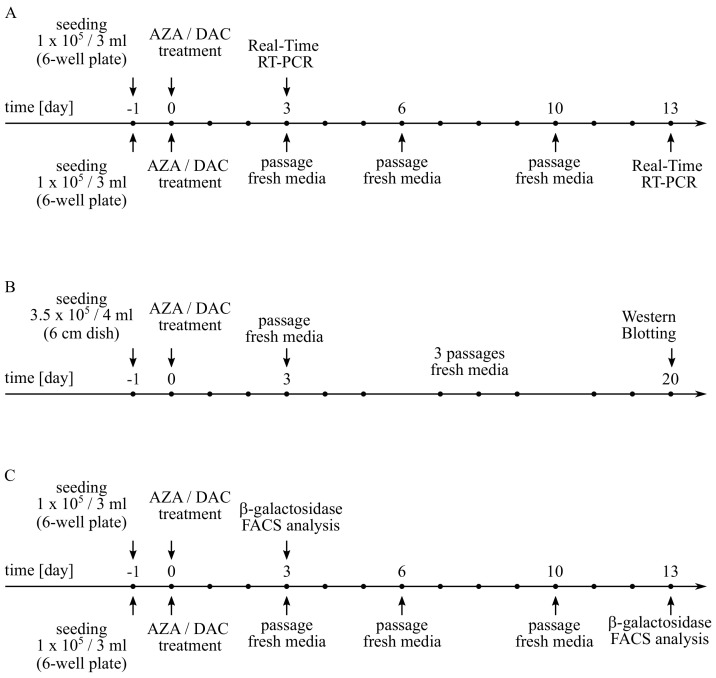
Detailed scheme of treatments for (**A**) gene expression assay, (**B**) 20 day long western blotting and (**C**) assesment of activity of SA-β-galactosidase.

The DLD-1, HT-29, and RKO cells were seeded at a density of 1 × 10${}^{5}$ cells/3.5 mL (9.5 cm${}^{2}$), the next day (day 0) decitabine (1 µM) or azacytidine (4 µM) was added, then the cells were cultured for 10 days (first passage after 3 days) and stained with 1 µM CellTrace Far Red (Invitrogen) for 20 min under standard cell culture conditions and seeded at a density of 4 ×$10^{4}$ cells/1 mL (1.9 cm${}^{2}$) to measure cell proliferation on day 13. The cells were collected by BD FACSCalibur flow cytometer (Becton Dickinson, Franklin Lakes, NJ, USA) and the data were analyzed in Flowing Software 2.5.1 (Turku Centre for Biotechnology). For detailed treatment schedule, see Figure A7. The results are shown as a percentage of cells with inhibited proliferation relative to control (untreated cells). Untreated-unstained cells and untreated cells stained on the day of analysis were the additional controls.

### 2.8. Real-Time RT-PCR

The DLD-1 and HT-29 were seeded similarly to point 2.6. For detailed schedule, see Figure A7A. RNA was isolated using TRI Reagent Solution (Invitrogen) according to the manufacturer’s protocol and quantified by NanoDrop 2000 spectrophotometer (Thermo Scientific, Waltham, MA, USA). Genomic DNA was removed using DNase I (Thermo Scientific, Waltham, MA, USA) according to the manufacturer’s protocol. Reverse transcription was performed using Maxima First Strand cDNA Synthesis Kit for RT-qPCR (Thermo Scientific, Waltham, MA, USA) according to the manufacturer’s protocol. Real-time PCR was performed using DyNAmo Flash Probe qPCR Kit (Thermo Scientific, Waltham, MA, USA) and TaqMan Gene Expression Assays (Applied Biosystems, Thermo Fisher Scientific, Waltham, MA, USA) for RNA18S (#Hs99999901_s1), GAPDH (#Hs02758991_g1), CDKN1A (#Hs00355782_m1), CCND1 (#Hs00765553_m1), MDM2 (#Hs00540450_s1), MYC (#Hs00153408_m1), GLB1 (#Hs01035168_m1), and CDKN2A (#Hs00923894_m1). The reaction was performed on ViiA 7 Real-Time PCR System (Applied Biosystems) and the reaction conditions were as follows: 2 min at 50 °C, 10 min at 95 °C, 40 cycles of 15 s at 95 °C and 1 min at 60 °C. The data were analyzed by comparative $\Delta \Delta C_{T}$ method in QuantStudio 1.3 (Applied Biosystems). GAPDH was used as a reference gene for CDKN2A, whereas RNA18S was used as a reference gene for the others. The results are shown as a difference in target gene expression in treated cells relative to control (untreated cells).

### 2.9. SA-β-Galactosidase Activity

The HT-29, DLD-1 and HCT116 cells were seeded as described in point 2.6. At the end all cells were fixed with 4% paraformaldehyde for 10 min at RT, then stained with CellEvent Senescence Green Detection Kit (Invitrogen, Thermo Fisher Scientific, Waltham, MA, USA) for 2 h at 37 °C. The cells were collected and analyzed by BD LSRFortessa flow cytometer and Flowing Software 2.5.1. The results are expressed as mean fluorescence with subtracted background (untreated cultured cells). For detailed schedule, see Figure A7C.

### 2.10. Histochemical SA-β-Galactosidase Staining

The HT-29 cells were seeded and treated as described in point 2.6. At the end, the cells were stained on a culture 6-well plate with the Senescence Cells Histochemical Staining Kit (Sigma-Aldrich) according to the manufacturer’s instructions, with an overnight incubation in a Staining Solution. The cells were analyzed the next day. Microscopic images were taken in the bright field of Olympus CKX53 Inverted Microscope by MoticamBTU camera.

### 2.11. Statistical Analysis

The data are expressed as means ± standard deviations from at least 3 independent experiments. Outliers were identified based on the median absolute deviation. Two-tailed Welch’s *t*-test was used to compare the means of 2 independent samples, and *p* < 0.05 was considered significant. The calculated *p*-values are shown as: ns (*p* > 0.05), * (*p* < 0.05), ** (*p* < 0.01), *** (*p* < 0.001). In real-time RT-PCR, relative gene expression differences < 1.41 were considered non-significant.

## 3. Results and Discussion

To focus on the comparison of DNA-mediated effects of these twin 5-aza-modified analogs of cytosine, we used 4-times higher concentrations of azacitidine than decitabine. This ratio was chosen because only 10–35% of azacitidine is incorporated into the DNA, unlike 100% of decitabine [21,24]. Additionally, the 4:1 ratio reflects the differences of doses used in recommended therapeutic regiments [12].

**Figure 1:**
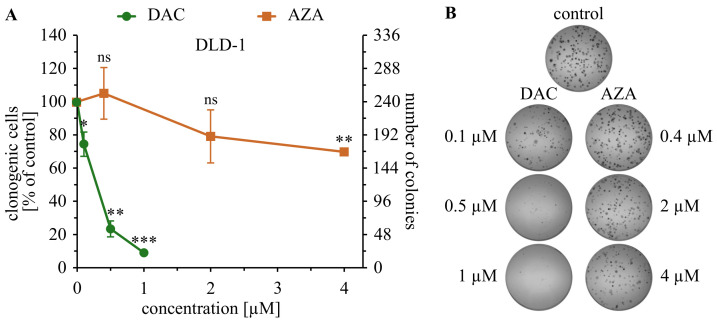
Decitabine (DAC), but not azacitidine (AZA), inhibits the growth of DLD-1 colorectal cancer cells in a clonogenic assay. (**A**) Average ± standard deviation of colonies number are shown on the graph; ns—not significant, *p* > 0.05; * *p* < 0.05, ** *p* < 0.01, *** *p* < 0.001 in *t*-Welch test. (**B**) representative photographs of plates with the colonies.

**Figure A8:**
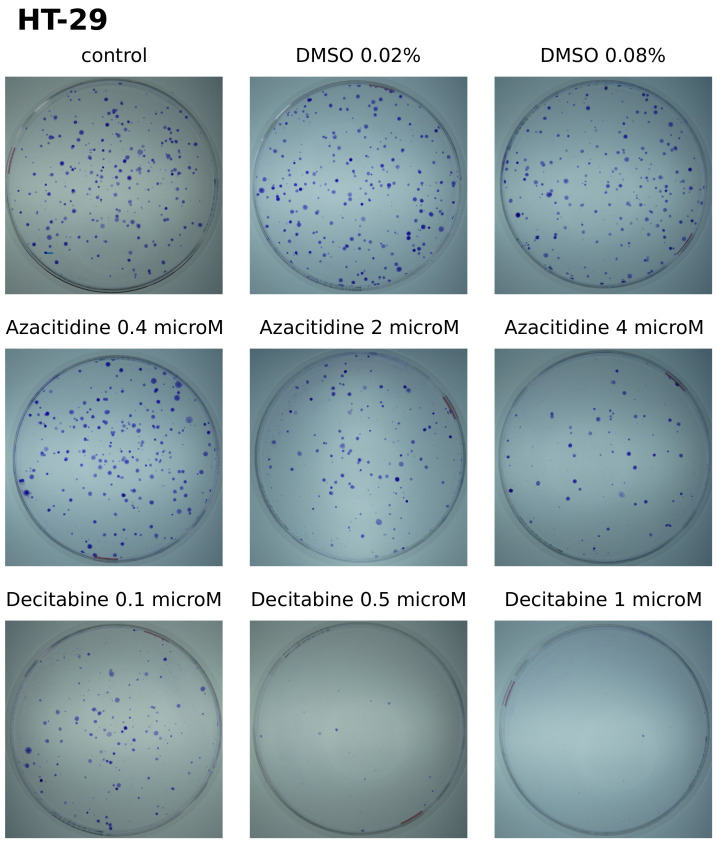
Clonogenic assay of HT-29 cells.

**Figure A9:**
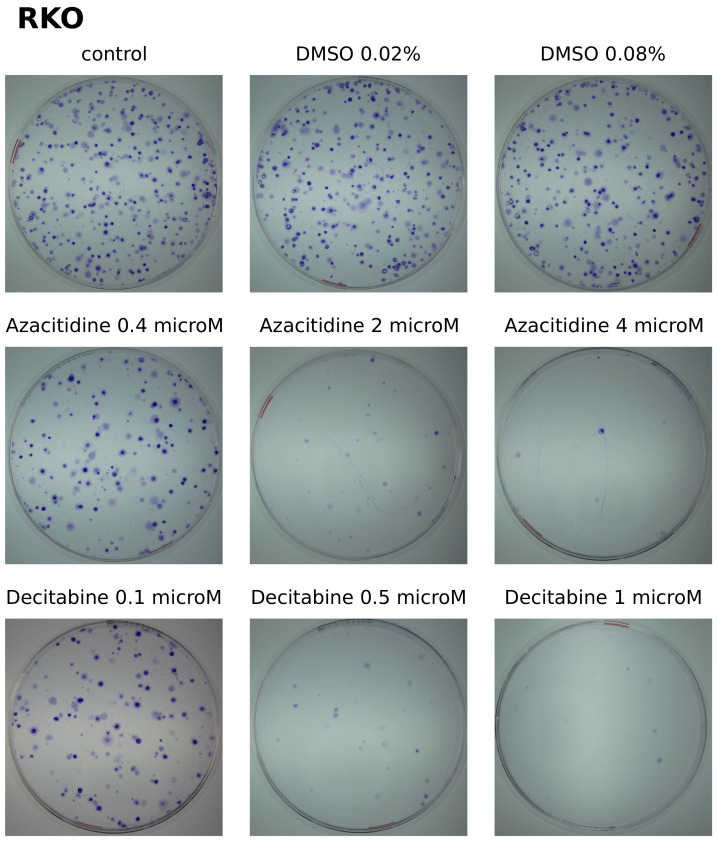
Clonogenic assay of RKO cells.

The clonogenic assay, in contrast to the short-time assays, allows the observations of anti-tumorigenic effects that extend beyond the immediate cytotoxicity. In some experimental set-ups, it may demonstrate the capacity for self-renewal of tumor cells population. We tested the effectiveness of azacitidine or decitabine against DLD-1 colorectal cancer cells in a clonogenic assay. We observed a strong inhibition of growth of the DLD-1 cells by decitabine (Figure 1). Minor inhibition of the cell growth was noticed even in 0.1 µM concentration, and inhibition was almost complete at 1 µM. This should be contrasted with the little activity of azacitidine, as 4 µM of azacitidine inhibited colony formation on level similar to 0.1 µM of decitabine. Lower activity of azacitidine can’t be explained merely by about 4 times lower incorporation of the drug into DNA. This result clearly demonstrates the minimal anti-clonogenic activity of azacitidine against DLD-1, contrasting with strong effect of decitabine. When HT-29 and RKO cells were tested, similar differences are observed (Figure A8 and Figure A9). The success of cancer cell to grow into a colony in the clonogenic assay partially depends on the direct susceptibility to cell death induced by the drug. In this regard, the wider panel of colorectal cancer cells have been selected for observation of immediate cytotoxicity induced by decitabine or azacitidine.

**Figure 2:**
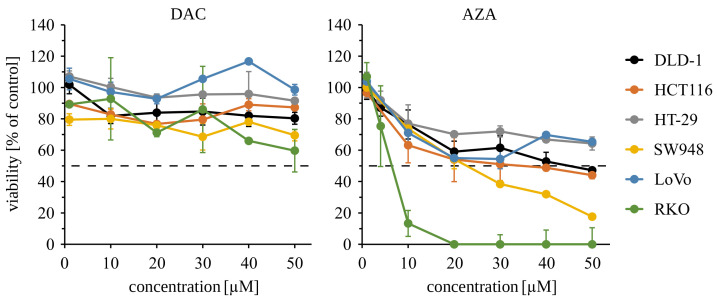
Azacitidine (AZA), but not decitabine (DAC), impairs the viability of several colorectal cancer cells in 3 days long MTS assay. Average viabilities and standard deviations of viabilities are shown on the graph. Cells treated with 0.01% of DMSO (solvent) were set as a 100% control. The dashed line represents 50% viability.

We observed dose-dependent cytotoxicity of azacitidine in all tested cell lines (Figure 2). The RKO cells were the most sensitive (IC_50_ = 5.6 µM), while HT-29 and LoVo cell lines were the most resistant (IC_50_ > 50 µM). In contrast, decitabine at 1 µM or even 50 µM concentration never reduces viability below 50% (Table 1). These results demonstrate the initial cytotoxicity of azacitidine but not decitabine. Despite some variability of sensitivity among the tested cell lines, disparate outcomes of short 72 h treatments with decitabine and azacitidine are clear. These results indicate that the initial cytotoxicity of azacitidine is not sufficient for inhibition of growth of cancer cells in a clonogenic assay. At the same time, the lack of initial toxicity of decitabine does not exclude its prolonged cytotoxic effect on colorectal cancer cells.

**Figure 3:**
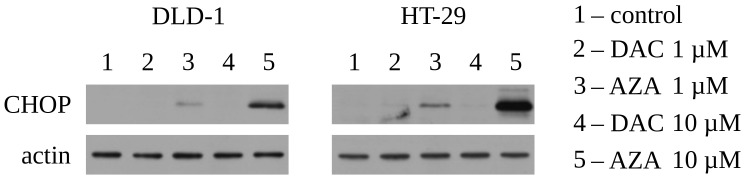
Azacitidine (AZA), but not decitabine (DAC), induces expression of CHOP, an ER stress marker on DLD-1 and HT-29 cells. Analysis was performed 20 h after treatment with specified drug. Representative blots are shown. The uncropped blots are presented on Figure A10 and Figure A11.

Next, we ask about the reasons for the initial toxicity of azacitidine. As it was previously mentioned, most of the azacitidine is rapidly incorporated into newly synthesized RNA. Then, it can interfere with transcription or translation processes [26,41,42]. The disturbances in both processes can lead to endoplasmic reticulum (ER) stress, reflected by expression and activation of CHOP transcription factor and to apoptosis. In this regard, we asked if azacitidine or decitabine can cause ER stress in DLD-1 or HT-29 cells. Indeed, treatment with azacitidine led to the concentration-dependent induction of CHOP expression (Figure 3). It indicates triggering of the ER stress response, which could lead to viability loss. These findings support previous reports suggesting that RNA-mediated and/or ER-related effects of azacitidine are the primary source of its cytotoxicity and anti-cancer activity, accordingly to preferential incorporation of azacitidine into RNA [25,42,43]. Consequently, treatment with decitabine doesn’t induce any expression of CHOP.

**Figure 4:**
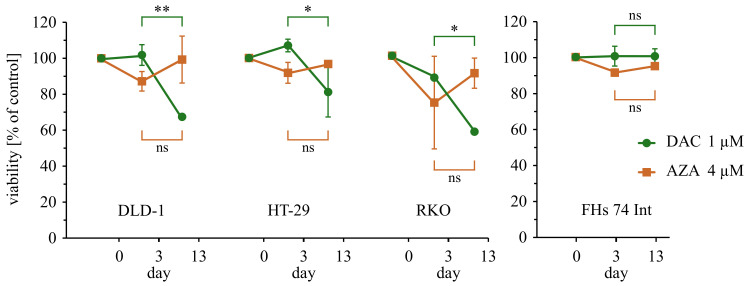
Comparison of viabilities of colorectal cancer cells on 3rd and 13th-day post-treatment with decitabine (DAC) or azacitidine (AZA). Average viability ± standard deviation are shown on the graph, ns: non-significant, *p* > 0.05; * *p* < 0.05; ** *p* < 0.01.

Beyond the direct initial cytotoxicity, the clonogenic assay tests survival and proliferation of cells separated from each other. That phenomenon is a matter of more subtle and long-term activities of drugs. In the case of epigenetic drugs, that poses genom-wide activity, these long-term should dominate the drug activity. In that matter, we studied the delayed consequences of a single treatment with 5-aza-modified cytosine analogs. We extended viability experiments up to 13 days, which were performed on DLD-1, HT-29, and RKO cell lines (Figure 4). During that additional time, decitabine-treated cells underwent significant viability loss, while azacitidine-treated cells were unaffected. To provide the context of normal tissue, we have tested the outcome of both treatments on the normal small intestine epithelial cell line FHs 74 Int. Interestingly, any of the drugs did not exert significant cytotoxicity on the 3rd nor 13th day of the treatment. Low toxicity of drugs can be attributed to insufficient dose, slow proliferation rate and metabolic activity of normal cell lines compared to cancer cells. Especially, small proliferation rate may result in decreased incorporation of the drugs to nucleic acids, and in consequence, prevents cytotoxicity. Nevertheless, the low toxicity to the normal cells should be regarded as an important advantage in the context of anti-cancer therapy.

**Figure 5:**
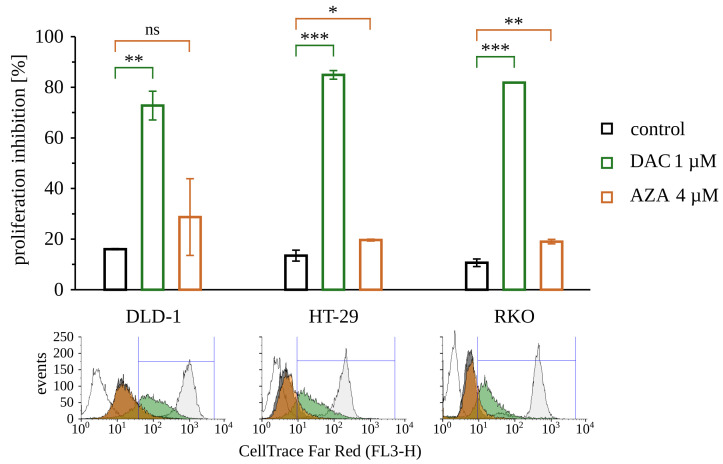
Inhibition of proliferation by decitabine (DAC) or azacitidine (AZA) on the 13th day after treatment. Average percentage of cells with inhibited proliferation ± standard deviation are shown on the graph; ns: non-significant, *p* > 0.05; * *p* < 0.05; ** *p* < 0.01; *** *p* < 0.001 (*t*-Welch test). Below, representative histograms are shown. Empty histogram—negative control, unstained cells; gray histogram—cells stained on day of the measurement, positive control; black histogram—untreated control, green histogram—cells treated with decitabine (DAC), orange histogram cells treated with azacitidine (AZA).

The viability loss observed on the 13th day after treatment with decitabine may arise from cell death and inhibition of proliferation. To assess proliferation rate, we choose the assay that is based on the dilution of fluorescent dye with every cell division. CellTrace Far Red fluorescent probe was applied on the 10th day after treatment with 5-azacytosine analog and analyzed 72 h later, on the 13th day (Figure 5). As expected, the dye was diluted the most in untreated cells, while cells treated with decitabine keep markedly more of the dye. It indicates that decitabine-treated cells underwent a fewer number of divisions. At the same time, the fluorescence intensity of cells treated with azacitidine was consequently only slightly higher than the fluorescence of the untreated cells.

**Figure 6:**
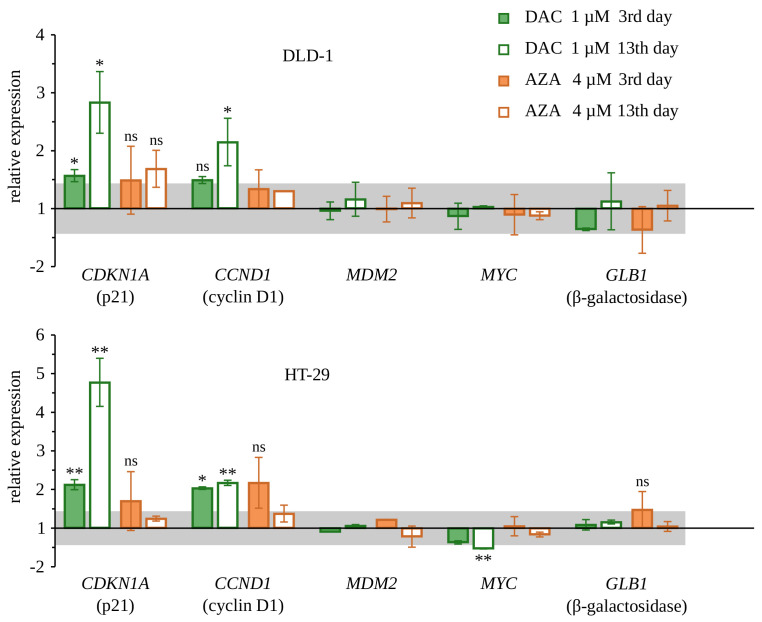
Expression of CDKN1A, CCND1, MDM2, MYC, GLB1 genes in DLD-1 or HT-29 cells upon treatment with decitabine (DAC) or azacitidine (AZA). Real-time RT-PCR analysis. Untreated cells served as a control. Average RQ ± standard deviation are shown on the graphs; ns: non-significant, *p* > 0.05, * *p* < 0.05, ** *p* < 0.01, (*t*-Welch test). Range of expression changes by less than half of the PCR cycle was marked as gray and regarded as insignificant.

**Figure 7:**
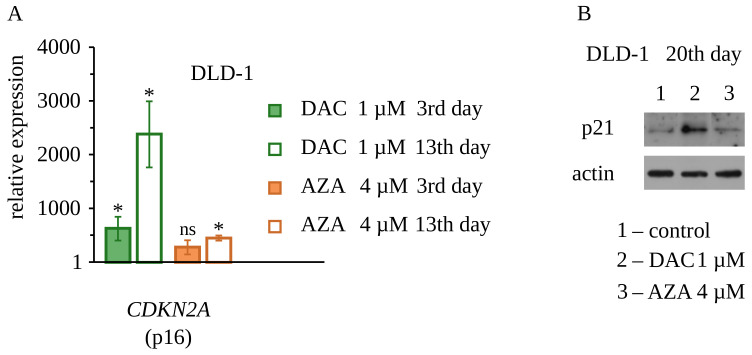
Expression of cell cycle inhibitors CDKN2A gene and p21 in DLD-1 cells upon treatment with decitabine (DAC) or azacitidine (AZA). (**A**) Real-time RT-PCR analysis. Untreated cells served as a control. Average RQ ± standard deviation are shown on the graphs; ns: non-significant, *p* > 0.05, * *p* < 0.05, (*t*-Welch test). Range of expression changes by less than half of the PCR cycle was marked as gray and regarded as insignificant. (**B**) Western blot analysis of p21 expression on the 20th day after exposure to DAC or AZA. Representative blots are shown. The uncropped blots are presented on Figure A12.

To explore molecular events accompanying retardation of the proliferation of cells treated with decitabine, we decided to study the expression of two genes that are important negative regulators of the cell cycle: CDKN1A (coding p21—inhibitor of cyclin-dependent kinases 1 and 2), CDKN2A (coding p16—inhibitor of cyclin-dependent kinases 4 and 6), and the expression of two genes that are positive regulators of the cell cycle: MYC (coding MYC—major transcription factor of proliferating cells), MDM2 (coding MDM2—inhibitor of p53). Gene expression was measured by real-time RT-PCR on the 3rd and 13th day after treatment of DLD-1 and HT-29 cells with decitabine or azacitidine (Figure 6 and Figure 7). We found up-regulation of expression of both genes coding negative regulators of the cell cycle, p21, and p16, in cells treated with decitabine. Consistently with the results presented earlier, this up-regulation was more profound on day 13 after the treatment. Up-regulation of p21 in DLD-1 cells was also confirmed on a protein level by Western blotting 20 days after treatment with decitabine. Additionally, in HT-29 cells, there was a slight down-regulation of MYC expression on the 13th day. Azacitidine, in turn, only increased the expression of the p16-encoding gene on the 13th day and did not affect the expression of genes encoding p21 and MYC. Importantly, CDKN2A coding p16 was not expressed in untreated DLD-1 cells, which is consistent with the previous report and results from promoter methylation of this gene [44]. It is worth mentioning, that decitabine despite that was used in a lower dose than azacitidine, induced stronger CDKN2A expression. None of the drugs changed the expression of MDM2. These results suggest that up-regulation of p21 and/or p16 may be involved in the inhibition of cell proliferation by decitabine. It can be discussed if observed up-regulation of CDKN1A and CDKN2A genes originate directly from demethylation of these particular genes (DNA region/sequence specific process) or these are the result of stress response to the broad deregulation of genes expression caused by incorporation of decitabine into DNA. Some studies have shown that CDKN2A is silenced by methylation in DLD-1 cells, and thus can be reexpressed by methylation inhibitors [44].

Little is known about the duration of the effect of DNA methylation inhibitors on gene expression. Mossman et al. have shown that some genes that were hypo-methylated in CpG sites adjacent to the transcription start site remained expressed even 10 days post-decitabine treatment [39]. Similarly, it was observed that even as non-tumor methylation is promptly regained, the tumor-specific hypermethylation recovers more slowly [40]. This strongly suggests the temporal nature of demethylation and the re-expression of most genes. Additionally, the incorporation of decitabine into DNA by DNA polymerase should be regarded as a stochastic process. It means that there should be no preference for specific genes [45]. In that circumstances, the alteration of gene expression should be viewed as an outcome of the broad deregulation of cell function caused by DNA methylation inhibitors.

**Figure 8:**
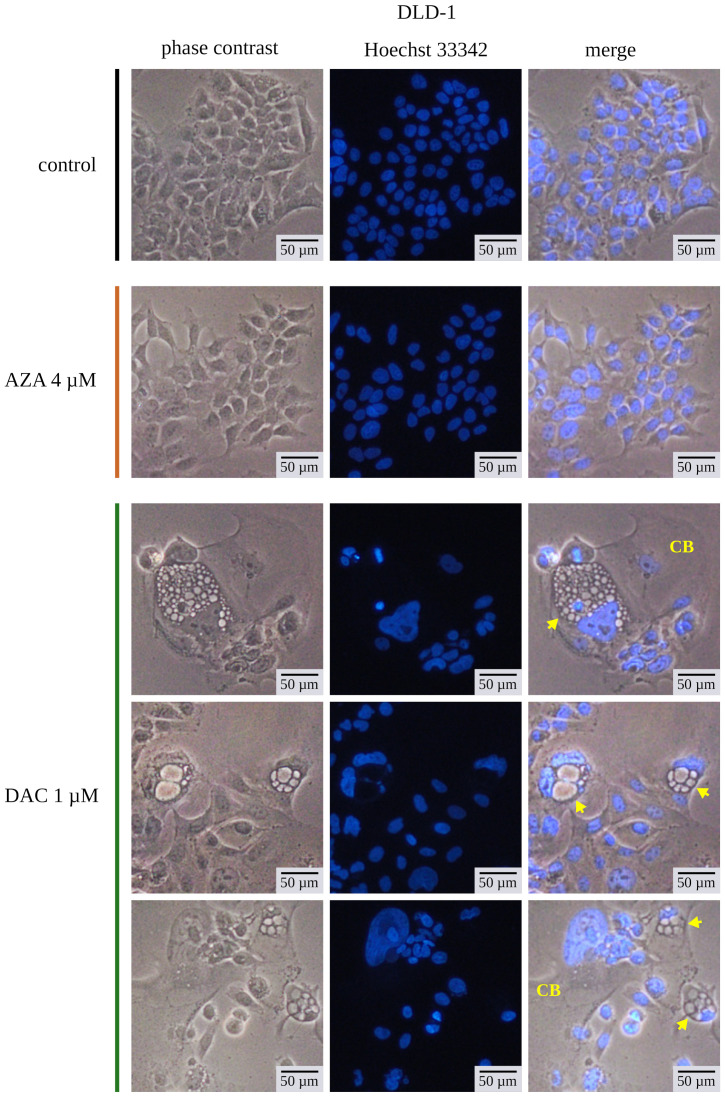
Changes of the morphology of DLD-1 cells on the 13th day after treatment with decitabine (DAC) or azacitidine (AZA). CB indicates cells with large bodies, and arrows indicate large intracellular vehicles. Representative microphotographs are shown.

**Figure 9:**
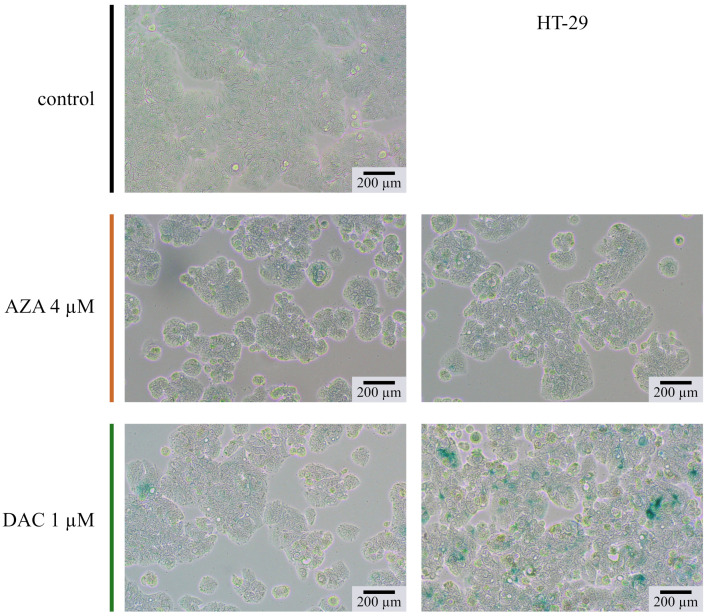
Cytohistochemistry based assay of SA-β-galactosidase in HT-29 cells on 13th day after treatment with decitabine (DAC) or azacitidine (AZA). Increase in green color indicates activity of SA-b-galactosidase. Representative microphotographs are shown.

**Figure 10:**
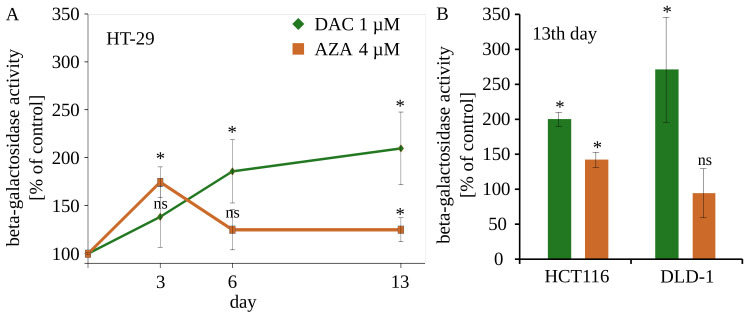
Activity of SA-β-galactosidase after treatment with decitabine (DAC) or azacitidine (AZA). (**A**) Time course of activity of SA-β-galactosidase in HT-29 cells. (**B**) Activity of SA-β-galactosidase in DLD-1 and HCT116 cells on the 13th day after exposure to DAC or AZA. Averages ± standard deviation are shown on the graph. ns: non-significant, *p* > 0.05, * *p* < 0.05 (*t*-Student test).

The experiments were complemented by contrast light inverted microscopy and fluorescent microscopy with Hoechst 33342 dye for chromatin visualization (Figure 8). We analyzed the morphology of the DLD-1 cells on the 13th day after treatment. Cells treated with 4 µM of azacitidine don’t differ from untreated cells. It again underlines limited delayed outcomes of azacitidine in colorectal cancer cells. Contrary, DLD-1 cells treated with 1 µM decitabine exhibited many morphological abnormalities. Enlarged cells with enlarged nucleus dominated the culture. Additionally, some cells contained many vesicles. Such phenotype may suggest that cells are undergoing cellular senescence [46], which is consistent with previous findings of Putri et al. on short time-treated MCF or U2OS cells [47,48,49]. The senescence is described as a cellular condition accompanied by enlargement of a cell, inhibition of proliferation, increased activity of SA-β-galactosidase, and secretory phenotype. The activity of the acid SA-β-galactosidase is a well-established marker of cellular senescence. Despite lack of induction of GLB1 expression (Figure 6) We found increasing over time activity of SA-β-galactosidase in HT-29 cells treated with decitabine (Figure 9). The azacitidine-treated cells underwent transient increase in b-galactosidase activity. Further decrease of activity can be due to decreasing portion of the cells that underwent senescence. The increased activity of SA-β-galactosidase was also observed on 13th day after treatment in HCT116 and DLD-1 cells (Figure 10). In all cases, the activity was stronger in decitabine-treated cells.

The other feature of the senescence is up-regulation of the CCND1 gene, coding cyclin D1 activator of cyclin-dependent kinases 4 and 6 [50,51], that is linked to inhibition of proliferation. We found up-regulation of CCND1 and proliferation inhibitors p21, p16 in the cells treated with decitabine, but not azacitidine, on the 13th day after treatment (Figure 6 and Figure 7). The important feature of cellular senescence is the complete block of the cell cycle. Yet, we still observed the dividing cells (Figure 5), which may suggest the occurrence of processes besides senescence. But, it should be noticed that proliferation inhibition and senescence should be regarded as secondary events, being a consequence of more deep perturbations of cell genome or metabolism. Additionally, we should regard the stochastic nature of the activity of 5-azanucleosides, there is no discrepancy in the non-uniform response of cell population to 5-azanucleosides [45]. Mossman et al. has shown induction of apoptosis in LoVo or SW480 upon treatment with 10 µM of decitabine, the effect was greatly reduced on the 13th day [39]. In the same studies, the significant viability loss observed on the 10th-day post-treatment with decitabine was interpreted as necrosis. Thus, the occurrence of other cell death modalities like necroptosis, paraptosis or autophagy is not excluded [52]. Nevertheless, in our studies we didin’t observe any signs of apoptosis upon treatment with decitabine. Contrary, our results strongly point out that senescence should be regarded as the main long-term outcome of transient exposition to decitabine. Decitabine can exert prolonged change of gene expression and durably affects the viability of cell cultures that finally leads to cell senescence [53]. Contrary, the outcomes of azacitidine treatment are transient, even though 4 times higher concentration was used in the experiments. The simplest explanation of the lack of prolonged activities would be that too small portion of drug incorporated to DNA in colorectal cancer cells. It could be due to the lower proliferation rate of colorectal cancer leukemia cells. Therefore, the majority of the azacitidine is then built-in into RNA and is responsible for immediate toxicity. While higher doses of azacitidine would emphasize the epigenetic activity of the drug, the immediate drug toxicity could predominate the overall effect of azacitidine. Likely, the initial stress response to RNA damage lags cell growth, prevents incorporation of the drug to DNA, and undermines the long-term epigenetic effects in some experiments.

This, along with recent findings that azacitidine could inhibit nonsense-mediated RNA decay [26], disrupts chromatin organization [24], interfere with RNA polymerase complex, inhibits ribonucleotide reductase [42], and affects methylation of tRNA [54] suggest that azacitidine should be regarded rather as a drug with novel RNA related activities. These reports along with our 13-day-long studies challenge the epigenetic activities of azacitidine in colorectal cancer cells. It is especially well demonstrated by the clonogenic assay (Figure 1). Single azacitidine treatment had very little impact on the growth of colorectal cancer cells; decitabine almost completely inhibited the growth of clonogenic cells in low 1 µM concentration. This is consistent with a recent study demonstrating decitabine-induced molecular re-programming of cancer cells, influencing the Wnt pathway and leading to reduction of cancer cell stemness [55]. It shows the potential of decitabine as a therapeutic that especially targets cancer stem cells, probably via epigenetic mechanism [56,57]. At the same time, we need to acknowledge that the anti-cancer effect of decitabine requires a relatively long time for development. It is important, especially in the context of combination therapy. It was shown that re-expression of CDKN1A [58] or other tumor suppressor genes [34] can restore or increase cancer cell chemo-sensitivity. Finally, there is growing recognition of the role of aberrant methylation in progress and development of CRC. The hipermethylation is a common feature of CRC cancer cells that affects a wide range of molecular pathways [59,60,61]. Interestingly, in some DAC-resistant MDS patients, response to AZA and vice-versa [62,63]. It shows that the consequences of disparate mechanism of DAC and AZA can be observed in clinics. Additionally, it was shown that TET2 mutations (a protein that takes part in reversing the cytosine methylation) enhance the treatment response of MDS patients to hypomethylating DAC, underlining its truly epigenetic activity [64]. Our study points out, that not all such drugs have equal long-term, probably epigenetic activity. There is a need for long-term evaluation of activity of a particular epigenetic drug before its introduction as an epigenetic modulator.

We have shown that the expression of genes like CDKN1A or CDKN2A was the highest on the 13th-day post-treatment. Accordingly, it was demonstrated by Hosokawa et al. that pretreatment is better than co-treatment when epigenetic drugs are used with other chemotherapeutics [65]. Similar conclusions can be drawn from the recent study of Cruijsen et al., where preconditioning with DAC increased the efficacy of non-myeloablative (NMA) conditioning in poor-risk AML patients [66]. Nevertheless, we need to be careful while drawing conclusions regarding clinical practice. But, long-term activity of decitabine and other epigenetic drugs presents as an opportunity for bigger separation of doses in combination therapy that could ease the unwanted toxicity. It needs to be carefully considered in designing schedules of combination treatments [15,65].

Last, but not least, it must be noted that all discussed, short-term or long-term effects were observed on the cancer cells. Neither decitabine nor azacitidine decreased the viability of normal intestine cells, indicating that at least some effects of the drugs are more profound in cancer cells.

## 4. Conclusions

1. Decitabine has limited direct and short-term cytotoxicity. Extension of observation of single-dose-treated cells up to 20 days reveals profound abnormality of morphology, abrogation of proliferation, and accompanying re-expression of CDKN1A and CDKN2A; all its effects leads mainly to induction of cellular senescence; 1 µM is sufficient for almost complete inhibition of clonogenicity. Such a long observation time is necessary for the development of outcomes of one-time decitabine exposure. It indicates an urgent need for careful design of experiments aforethought to trace the effects of its epigenetic manipulations.

2. Azacitidine decreases the viability of colorectal cancer cells in a 3-day-long assay, which is accompanied by induction of CHOP (indicating ER stress). At the same time, the prolonged, presumably epigenetic, activity of azacitidine is minute. On the 13th day after treatment, cell morphology is unaffected, the viability of cells and growth inhibition in the clonogenic assay are unsatisfactory. These results challenge the epigenetic activity of azacitidine in colorectal cancer cells.

3. Based on differential short-term and long-term effects on cancer cells, decitabine and azacitidine, despite being chemical analogs, should not be regarded as analog drugs.

## Figures and Tables

**Table 1:** IC_50_ [µM] of decitabine (DAC) or azacitidine (AZA) in various colorectal cancer cell lines.

	DLD-1	HCT116	HT-29	SW948	LoVo	RKO
DAC	>50	>50	>50	>50	>50	>50
AZA	44.9	31.7	>50	21.9	>50	5.6

**Figure A6:**
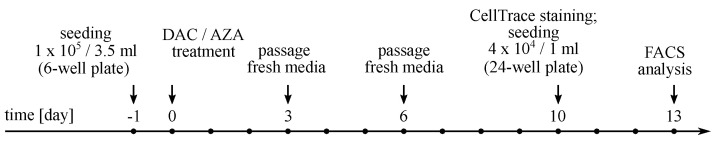
Detailed scheme of treatments for proliferation assay.

**Figure A10:**
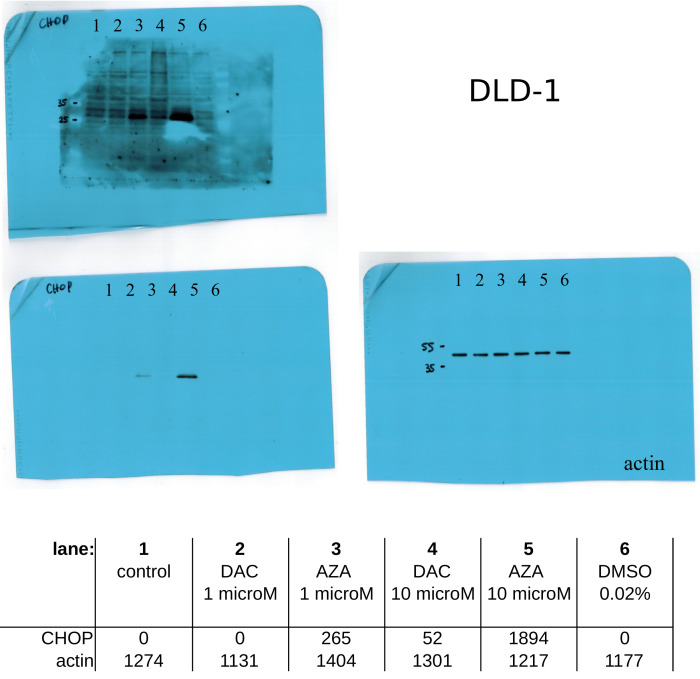
Whole western blots of CHOP assayed on DLD-1 cells (Figure 3), along with densitometric quantification.

**Figure A11:**
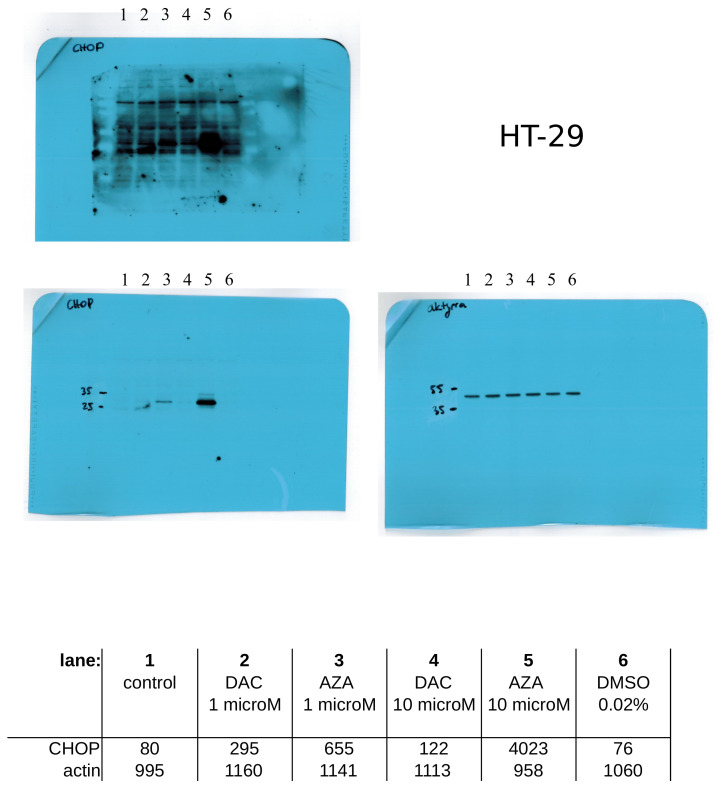
Whole western blots of CHOP assayed on HT-29 cells (Figure 3), along with densitometric quantification.

**Figure A12:**
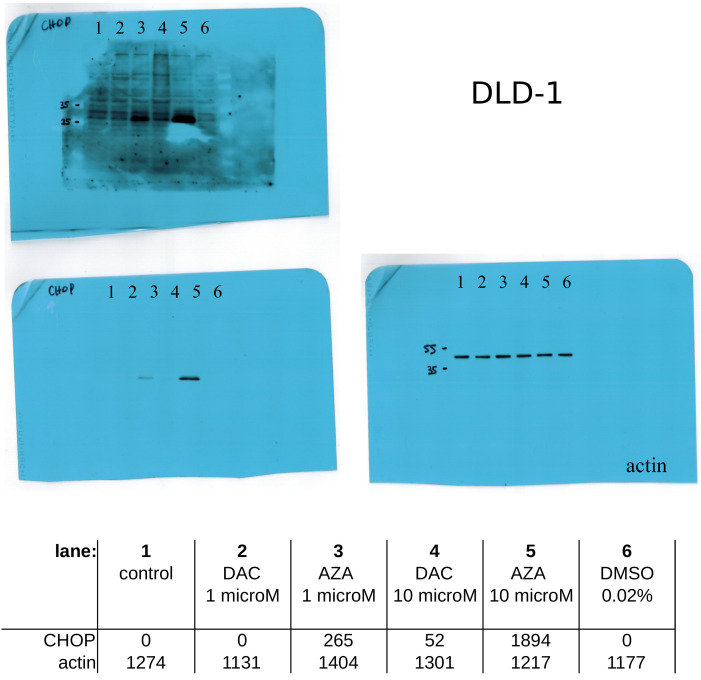
Whole western blots of p21 assayed on DLD-1 cells (Figure 7B), along with densitometric quantification.

## Data Availability

Data aviaable on request from corresponding author.

## Sample Availability

Not applicable. The study was performed using commercially available materials and reagents.
